# The weight of pupils’ schoolbags in early school age and its influence on body posture

**DOI:** 10.1186/s12891-017-1462-z

**Published:** 2017-03-21

**Authors:** Anna Brzęk, Tarja Dworrak, Markus Strauss, Fabian Sanchis-Gomar, Ibtissam Sabbah, Birgit Dworrak, Roman Leischik

**Affiliations:** 1 Department of Kinesiology, School of Health Sciences, Medical University of Silesia, Ul. Medyków 12, 40-754 Katowice, Poland; 2 Lectureship Prevention, Health Promotion, Department of Biology and Environmental Sciences, Carl von Ossietzky University, Oldenburg, Germany; 3 Lectureship Prevention, Health Promotion, University Witten/Herdecke, Faculty of Health, School of Medicine, Hagen, Germany; 4 Leon H. Charney Division of Cardiology, New York University School of Medicine, New York, USA; 5 Department of Physiology, Faculty of Medicine, University of Valencia and Fundación Investigación Hospital Clínico Universitario de Valencia, Instituto de Investigación INCLIVA, Valencia, Spain; 6 Faculty of Public Health, Lebanese University, Saida, Lebanon

**Keywords:** Weight of the school bag, Body posture, Pupils, Asymmetry

## Abstract

**Background:**

Postural development progresses through a series of stages (growth spurts, development of balance and coordination, postural stability) which occur when children are at school age. The reduction in the level of physical activity, increased body weight, overloaded school bags, asymmetry of the backpack straps, the method of putting on and taking off the backpacks and increased usage of electronic devices have negative side effects such as bad body posture habits.

**Methods:**

A prospective cohort study in the group of 155 pupils at early school age 7–9 years old has been conducted. Examinations have been conducted twice: first, at the beginning of the school year (initial examination) and second – after 10–11 months (final examination). Age, gender, BMI, weight of school bag carried to school and the length of straps have been assessed. Body posture measurement (using Adams’ test), the evaluation of the plumb line deflection from the gluteal cleft, the angle values of kyphosis and lordosis (according to Dobosiewicz methodology) and the pelvis and shoulder blades position (using a ruler and pediscoliometer) have been also measured.

**Results:**

The mean weight of a school bag in the initial study was 6.3 ± 0.8 (range between 4,7 and 9 kg). A tendency to carry slightly heavier school bags was noted in boys (6.7 vs. 5.9 kg; *p* = 0,00001). This tendency has linearly changed with age (*R* = 0.68; *p* < 0,001). In 3.2% of all school bags of children, weights exceeded norms with regard to the weight of the pupil. The increase of torso rotation exceeding norms was observed in 35.3% of girls (mean 2.7 ± 1.2) and in 60.9% of boys (mean 2.3 ± 1.3). The increase of kyphosis angle was noted in 48.5% of girls and in 36.8% of boys. The difference of straps length had a significant influence on the increase of rotation in upper thoracic spine, thoracolumbar junction and it also had influence on the decrease of lumbar lordosis in the group of girls.

**Conclusions:**

Differences in the weight of school bags after one school year have influenced changes in body posture abnormalities, especially in rotation parameters. Backpack straps asymmetry was noticeably stronger in the group of girls and the difference between braces may have an impact on some posturometric parameters. Lack of proper backpack lifting skills tends to create programs and training systems in this regard.

## Background

Postural development progresses through a series of stages that occur while children are of school age [1]. During this period, dynamic and rapid changes in growth are observed [2, 3]. The first of these periods, discussed in this report, coincides with the start of a primary education of the child. The second period includes puberty, when the child is between 13 and 15 years old [1, 4]. The transition to more sedentary lifestyle in a world of sedentary behaviors [5, 6] (for example, several hours of sitting during learning at school and at home, watching TV, and using PC [7, 8]) and the reduction in physical activity [9, 10] in favor of extra activities performed mostly in sitting positions, such as learning foreign languages or playing musical instruments [11] might lead to obesity [12–15]. All of these changes are negative factors that lead to the development of postural disorders. Young children adapt to these changes, although not always in an appropriate and favorable way [16].

On the one hand, posturogenesis is affected by the characteristics of growth spurt period, while on the other hand, posturogenesis is also affected by improper static body positions, e.g. in classrooms which are not spacious enough [16]. Important elements which should also be taken into consideration are school bag weight, quality and fit (i.e., shoulder strap size adjustment, strap asymmetry and the method of putting on and taking off the backpack). Biomechanically, the center of gravity acts on the support quadrangle and ensures stability in standing position [17]. Static and dynamic postural sways are particularly important elements while carrying a school bag. Young children adopt adaptive strategies [18] very quickly, compensating to align particular body segments in relation to one another and these adjustments may lead to postural abnormalities, e.g. within the sagittal plane over a long period of time [19, 20]. 90% of scoliosis are idiopathic – the causes are not known. For young children especially with bad posture or spine deformities, the care on ergonomic behavior is much more important [21, 22]. The worst case scenario occurs when a postural abnormality of idiopathic origin is neglected because these modifiable factors cannot be controlled or changed [23].

Research in this area show that although the average loads vary greatly between studies, the majority of reports indicate that the loads carried by pupils are greater than the recommended limits [24, 25]. Some researchers hypothesize that the use of heavy backpacks may contribute to the high reports of back pain, bad body posture and it is a frequent cause of discomfort for schoolchildren [26]. Moreover, asymmetry in school bag straps (most often adjusted to be too short) causes muscle imbalance. If the school bags of inactive pupils are overloaded, then the risk of serious postural abnormalities greatly increases, as these children are still within the critical period of posturogenesis [1, 4, 27]. Unfortunately, according to the Chief Sanitary Inspectorate, the majority of pupils carry overloaded school bags. On August 25, 2009, the Minister of National Education Regulation approved a maximum load for younger pupils equal to 10–15% of their body mass [Journal of Laws, No. 130, item 1130] [28]. Children in early primary school are not conscious of their health or the potential threat of postural abnormalities because they are not informed. Even if they are informed in this topic, following the principles of ergonomics is extremely difficult and it takes time to modify the pattern encoded in the central nervous system.

Habits which they exhibit are often exemplifications of improper behaviors of their older schoolmates and observations of the world around them, because they imitate to gain acceptance [16]. The problem of overloaded school bags was a very important question which emerged while the hypothesis for this study was being created. This problem has been identified but not yet solved. The research presented in this study is only a portion of a larger scientific project.

Following questions have been posted:

1. What is the average weight of a school bag carried by a child in early years of primary school and how does it change during the school year?
2. Are the straps of a school bag symmetric and properly adjusted?
3. How do posturometric parameters change among children in early primary school and on which factors do these potential changes depend?
4. To which degree does school bag weight correlate with posturometric results?
5. How important is the weight assessment in prophylactic examinations?
6. Are there any differences between girls and boys?

The aim of this study was to analyze if the change in school bag weight has been associated with the change of children body posture and to describe how school bag characteristics (straps/weight) may affect the back health.

## Methods

This was an observational study in which a group of pupils in primary schools in the region of Silesia in Poland has been examined. Examinations have been conducted twice for the same group of children: first, at the beginning of the school year (initial examination), second – after 10 months (final examination). The sample size was calculated using the sample size calculation for proportions. Assumed prevalence of posture disorders of 38–39% as it was reported in the study of Pokrywka et al., 2011 [29] and margin error of 6% with a confidence level of 95% and a population size of 500 to give a sample size of 168 pupils [30] have been used.

The study included a group of 155 pupils between 7 and 9 years old (mean age = 7.6 ± 0.6). The study was based on the prevention study within the framework of a project under resolution No. KNW – 1 – 002/N/1/0 conducted by the Medical University of Silesia, involving the study of children, adolescents, their parents and teachers in the region of Silesia from 2011 to 2016. There were two subgroups according to sex: 68 (43.9%) girls and 87 (56.1%) boys. All of the pupils in both groups had proper body posture. Pupils were selected on the basis of body posture examination; if no irregularities or mild asymmetries (e.g., spatial body position within shoulder blades and shoulders) have been observed, they were included in the study. All of the children included in the study have been assessed by the first author during the initial examination under the same conditions, using classic tools and tests for body posture evaluation (suggested for use by SOSORT) [31]. Children were excluded from the study if they were diagnosed with scoliosis or if they revealed serious posture abnormality in three planes. These children were referred to an orthopedic specialist for further evaluation. Other exclusion criteria were as follows: below the age of seven or above the age of nine, regular training in after – school sport clubs, exemption from physical education classes because of frequent infections or orthopedic injuries and moving to school by car. 155 children were chosen for the final analysis. Remaining children were excluded from the study because of a “growth spurt” which has been detected in excluded children and could have influenced the results of the posture evaluation.

### Data collection

**Fig. 1:**
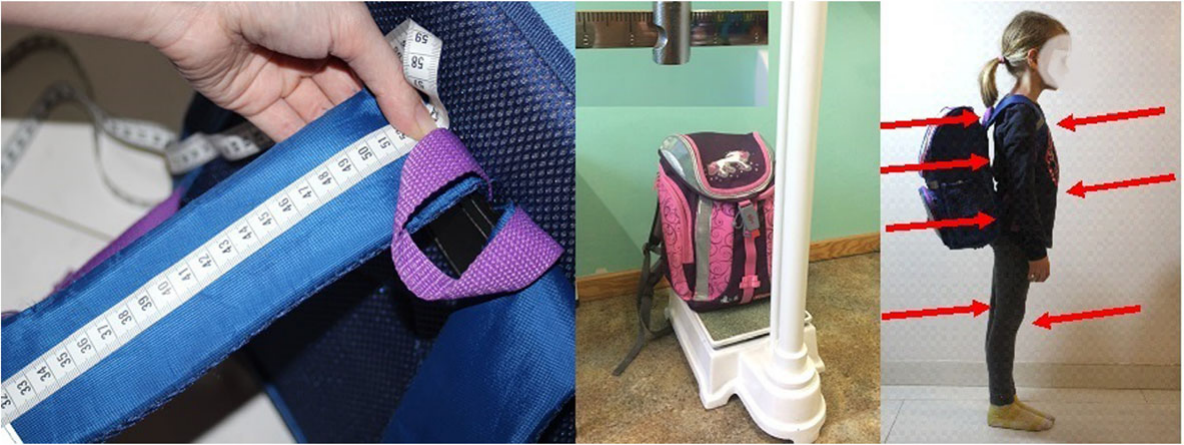
The assessment of straps lengths, school bag’s weight and school bags’ positioning on pupils’ back

In the examined group height and weight of the body have been measured and BMI has been calculated and further interpreted as a BMI percentile [32, 33]. School bag weight has been measured as a total value of two measurements during one school day and then repeated after 10 – month interval using a tared scale before the start of exams. Measurements have been carried out for the same group of children. As the examined pupils received seven lessons every day (i.e., from 8:30 to 14:50), no significant differences in school bag weight have been noted between particular days. The length of straps, appropriateness of the technique used to put on and take off a school bag and the symmetry of both straps have been also assessed. This investigation was a part of large project conducted by the first author connected with exemplifications patterns [23]. Children have been visually evaluated for the correctness of putting on and taking off backpacks. Trunk flexion (bending) or trunk extension and straight knees combined with trunk rotation (backpack positioned relative to the body on the right or the left side of the body) have been considered as erroneous. Raising a backpack through a squat (backpack set close to the body in the middle of the line) has been evaluated as correct. School bag weight (Fig. 1) has been measured on a tared medical scale. Lengths of straps have been measured in centimeters using centimeter tape.

Estimated weight of the school bags based on the recommendation of the Minister of National Education has been separately calculated for each pupil (maximum load for younger pupils equal to 15% of their body mass) and predicted value has been calculated as a percentage of exceeded weight of schoolbags of the pupils permissible for weight of the pupils.

To properly position a school bag on the back of the pupil, the middle of a bag was placed at the middle of the back of the pupil.

**Fig. 2:**
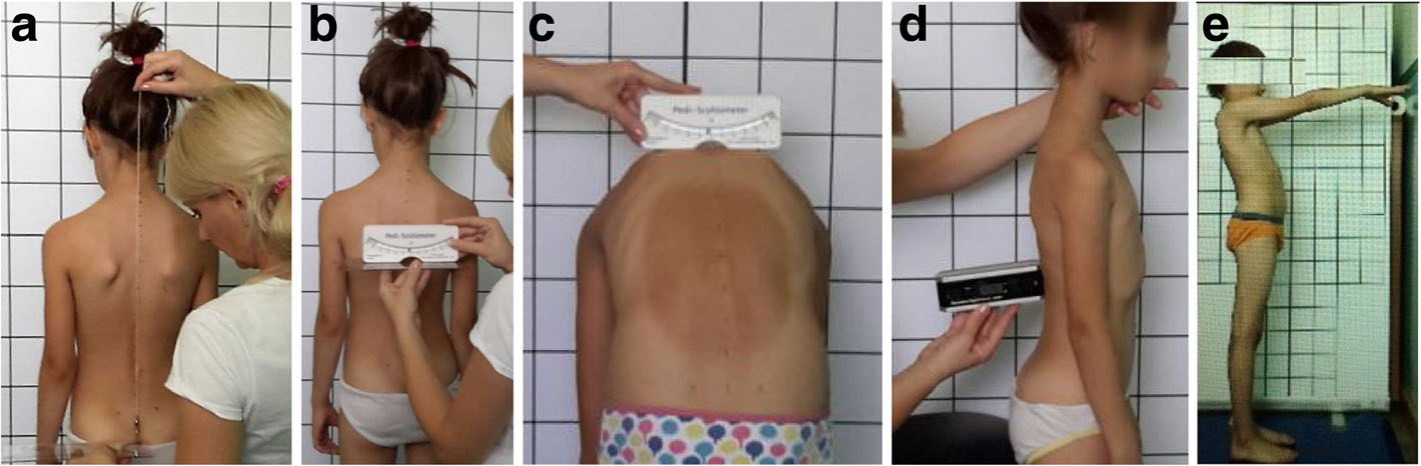
**a**, **b**, **c**, **d**, **e** The clinical study of the instruments use (**a**- the plumb line, **b**- the shoulder level, **c**- ATR, **d**- kyphosis and lordosis angle, **e**- Matthias test)

Body posture measurement has been performed according to the following methodology. Three measurements of the torso rotation angle were recorded with a Pedi-Scoliometer while the pupil stood in the Adam’s test position: in the peak of cervical kyphosis, in thoracolumbar junction and in the peak of lumbar lordosis. The highest torso rotation angle measurement from each level was recorded. In scoliometer examination, the angle of trunk rotation (ATR) has been adopted as a norm at the level of ≤ 3°. Based on the obtained results, the Hump Sum indicator was calculated to avoid a mistaken overdiagnosis of body posture abnormalities [34]. Next, the plumb line deflection has been evaluated starting from the gluteal cleft. The plumb line has been projected from the external occipital protuberance to the gluteal cleft, and its right or left deflection was expressed in centimeters. Subsequently, angles of kyphosis and lordosis have been measured according to the Dobosiewicz methodology [35] using a digital inclinometer (Saunders TMX – 127). Pelvis and shoulder blade positions have been measured using a ruler and a Pedi – Scoliometer (Fig. 2).

Body posture characteristics were described using following parameters:

1. Plumb line (projected from external occipital protuberance) falling down on gluteal sulcus – distance from gluteal cleft ≤ 0,5 cm (Figure No. 2a),
2. The level of shoulder blades position measured using a ruler and scoliometer ≤ 2^o^ Figure No. 2b),
3. Angle of trunk rotation (ATR) measured with scoliometer ≤ 3°for each spine section Figure No. 2c),
4. Thoracic kyphosis and lumbar lordosis angles in median plane measured using digital inclinometer, included in the range of values 24–36 °described by Dobosiewicz (Figure No. 2d)
5. Matthias test to evaluate postural muscle endurance during a minimum of 30 s (Figure No. 2e) [36].

Following factors were taken into account: school bag weight and school bag straps characteristics. Changes in school bags straps in the final examination – relative to a preliminary examination were recoded into three categories: stabilization, shortening and elongation. Body height, torso rotation angle, angles of kyphosis and lordosis, position of shoulder blades, deflection of the plumb line from the gluteal cleft and Hump Sum indicator were also taken into account. In addition, variables concerning unnecessary things in a school bag (but in opinion of pupils – „indispensable”) were also collected. The analyses of differences between examinations were performed with great attention to details. The results for both examinations (initial and final) have been analyzed independently and then compared (obtained differences between results were assessed and evaluated – dependency assessment).

### Statistical analysis

Data were described separately for two groups. Anthropometric parameters, clinical characteristics, body posture parameters and school bag parameters were expressed as mean values, standard deviations, minimum and maximum values and percentiles as appropriate. Categorical variables were expressed as percentage values. School bag weight as a percentage of body weight was computed by dividing weight of a bag by the weight of the child. Differences between groups were estimated via linear regression to answer following research question:” How do posturometric parameters change in children at early school age and on which factors do potential changes depend?”. Analyses were adjusted for age because most of the analyzed parameters were directly age – related and 95% confidence intervals (CIs) were calculated. Relationships between particular results have been searched sequentially, and the results are shown as a number (%), mean value (x) and standard deviation (SD). Subsequently, the differences between obtained parameters in both groups have been described. For data analysis following statistics methods have been used: Wilcoxon test (z) for continuous variables with non – normal distribution, T Student’s *t* test for continuous variables with normal distribution – to assess relationships between examinations, non – parametric characteristics test *χ* ^2^ and Rang Spearman test. Normal distribution has been verified by Kolmogorov – Smirnov test. Comparisons of statistical significance of differences between median values of parameters between groups were performed using ANOVA (analysis of variance). In order to assess the significance of differences in tested parameters at individual measurement points, a post – hoc Tukey test was used. Results with statistical significance lower than *p* < 0.05 were considered as statistically significant. Weighted Kappa Statistic was used as a measure of intra – examiner reliability and intra – class correlation coefficients were used as a measure of inter – examiner reliability for each method. A method of comparison analysis was performed to determine the 95% limits of agreement for all examiners. To avoid inter – tester variation, the same tester (first author) has carried out all tests in the same pupil. Statistica v.12 and Excel have been used for statistical analysis.

## Results

### Characteristics of the initial group

A preliminary review of the database did not reveal any characteristic results among examined groups aside from several unique individual results. Groups were homogenous, no statistically significant differences between girls and boys were noted in age, height or weight (Table 1). Inclusion criteria were based on the initial examination and required proper body posture or mild deviation from the norm for the age group (Table 2). After follow – up BMI in percentiles have not changed neither in girls (z = 1.57; *p* > 0.11) or in boys(z =0.27, *p* > 0.79).

**Table 1:** Initial characteristics of the examined group

Sex (F/M)	Age (year)		HEIGHT (centimeter)		WEIGHT (kilogram)		BMI (percentile)	
	Average	Range	Average	Range	Average	Range	Average	Range
Female (68)	7.4 ± 0.5	7–9	127.5 ± 4.3	120–137	25.2 ± 2.8	20.5–39.7	34.3 ± 18.5	3–97
Male (87)	7.7 ± 0.7	7–9	129.5 ± 5.0	123–142	27.1 ± 3.1	21.2–39.7	46.6 ± 31.6	5–97

*Abbreviations: F* female, *M* male, Mean ± SD (standard deviations) followed by min and max

**Table 2:** Comparison of pre – and post observational values of measured parameters by gender

Parameters	Sex	Before observation			During follow – up			*P*
		X ± SD	Range	95% CI	X ± SD	Range	95% CI	
Plumb – line anal cleft (cm)	*F*	0.3 ± 0.3	0–1	0.23–0.37	0.7 ± 0.5	0–2	0.6–0.8	<0.0001^a^
	*M*	0.3 ± 0.2	0–1	0.28–0.39	0.5 ± 0.5	0–2	0.4–0.6	<0.013^b^
Scapulae level (°)	*F*	0.9 ± 0.7	0–2	0.77–1.11	2.4 ± 0.7	0–3	2.2–2.6	<0.0001^a^
	*M*	1.0 ± 0.6	0–2	0.82–1.08	2.1 ± 0.7	0–3	1.9–2.2	<0.0001^a^
Kyphosis angle (°)	*F*	30.8 ± 4.4	22–36	29.69–31.8	35.1 ± 6.3	24–46	33.6–36.7	<0.0001^b^
	*M*	32.4 ± 4.0	21–36	31.52–33.22	34.9 ± 5.6	26–45	33.7–36.1	<0.0001^b^
Lordosis angle (°)	*F*	29.2 ± 4.5	24–36	28.1–30.28	32.6 ± 5.8	25–46	31.2–34.0	<0.0001^b^
	*M*	29.4 ± 4.8	24–36	28.34–30.4	30.6 ± 4.8	25–39	29.6–31.5	<0.006^a^
Angle of trunk rotation C_7_ – Th_1_ (°)	*F*	1.7 ± 0.9	0–3	1.5–1.95	2.0 ± 0.9	0–4	1.8–2.2	<0.014^a^
	*M*	2.0 ± 0.9	0–3	1.77–2.18	2.2 ± 1.0	0–4	2.0–2.4	<0.034^a^
Angle of trunk rotation Th (°)	*F*	2.1 ± 1.0	0–3	1.84–2.3	2.2 ± 0.8	1–5	2.0–2.4	>0.24^b^
	*M*	2.2 ± 0.9	0–3	2.0–2.4	2.6 ± 0.9	1–5	2.4–2.8	<0.002^a^
Angle of trunk rotation Th-L/L(°)	*F*	2.1 ± 0.8	1–3	1.9–2.27	2.3 ± 0.8	2–6	2.2–2.5	<0.04^b^
	*M*	2.1 ± 0.7	1–3	1.97–2.28	2.5 ± 0.8	2–6	2.4–2.7	<0.0001^a^
Sum of trunk rotation (HUMP SUM°)	*F*	2.3 ± 0.8	1–3	2.15–2.52	3.4 ± 1.3	2–7	3.1–3.7	<0.0001^a^
	*M*	2.6 ± 0.6	1–4	2.47–2.74	4.1 ± 1.5	2–8	3.8–4.4	<0.0001^b^
Mathiass Test (sek)	*F*	17.5 ± 9.6	1–30	15.19 - 19.86	17.1 ± 9.0	5–30	15.3–19.3	>0.29^b^
	*M*	17.3 ± 9.3	0–30	14.89 - 19.25	16.8 ± 8.1	4–30	15.1–18.6	>0.23^b^

Data are mean ± SD: Standard Deviation and Range; *P*-value according to ^a^Wilcoxon test (z) for continuous variables with non – normal distribution, ^b^T Student’s *t* test for continuous variables with normal distribution

*Abbreviations: F* female, *M* male, *C* cervical spine, *Th* thoracic spine, *L* lumbar spine, *Th – L* t horaco – lumbar spine

### School bags characteristics in the initial study and follow up

#### School bags weight

School bag weight in the initial examination ranged between 4.7 and 9.0 kg (mean weight = 6.3 ± 0.8 kg). There was a significant difference between girls and boys – boys had a tendency to have slightly heavier school bags than girls (z = 6.2, *p* < 0.00001). This tendency changed linearly with age (R Spearman = 0.8, *p* < 0.0001). School bags of boys in third grade were loaded by an average of 1.3 kg more than those of boys in first grade, and of 0.8 kg more than those of boys in second grade.

Among the examined group, school bag loads were related to the height of the pupils (R Spearman =0.3, *p* < 0.001) and weight (R Spearman =0.4, *p* < 0.001). The weight of the school bags of the pupils should range between 3.1 and 5.9 kg (mean weight = 3.9 ± 0.5 kg). Generally, in every examined case, school bag was heavier than the recommendation of the Chief Sanitary Inspectorate. For 64.7% of girls and 79.3% of boys, school bag weights exceeded the recommendation by more than 50% relative to the weight of the pupil (Table 3).

**Table 3:** Initial characteristics of the examined school bags of the pupils

Sex (F/M)		Weight of school bags (kg)		Estimated weight of school bags (kg)		Percentage of predicted value (%)		Excess weight of school bags (kg)		Shoulder straps length difference (cm)	
		X ± SD (Range)	Me	X ± SD (Range)	Me	X ± SD (Range)	Me	X ± SD (Range)	Me	X ± SD (Range)	Me
Female (68)		5.9 ± 0.5 (4.7–7.8)	5.9	3.8 ± 0. 4 (3.1–5.9)	3.7	157.0 ± 20.1 (94.1–253.6)	155.1	2.1 ± 0.7 (0.1–4.7)	2.04	2.8 ± 2.1 (0–9)	2.1
Male (87)		6.7 ± 0.9 (4.7–9.0)	6.8	4.06 ± 0.5 (3.2–5.9)	4.2	166.0 ± 24.8 (100.7–210.1)	167.2	2.6 ± 0.9 (0.1–4.6)	2.7	2.3 ± 1.8 (0–9)	3.0
*P - values* *Post-hoc*		0.0001		0.0001		0.01		0.0001		0.0001	
	{1/2} {2/1}	0.0001 0.0001		0.0001 0.0001		0.01 0.01		0.0001 0.0001		0.0001 0.0001	

Data are mean ± SD: Standard Deviation and Range; *P*-value according to ANOVA and Tukey *post hoc*

Estimated weight of school bags - calculated weight of school bags based on the recommendation of the Minister of National Education for each pupil separately (maximum load for younger pupils equal to 15% of their body mass)

Predicted value - calculated percentage of exceeded permissible weight of school bags of the pupils

*Abbreviations* **:** *F* female, *M* male, Mean ± SD (standard deviations) followed by min and max

**Fig. 3:**
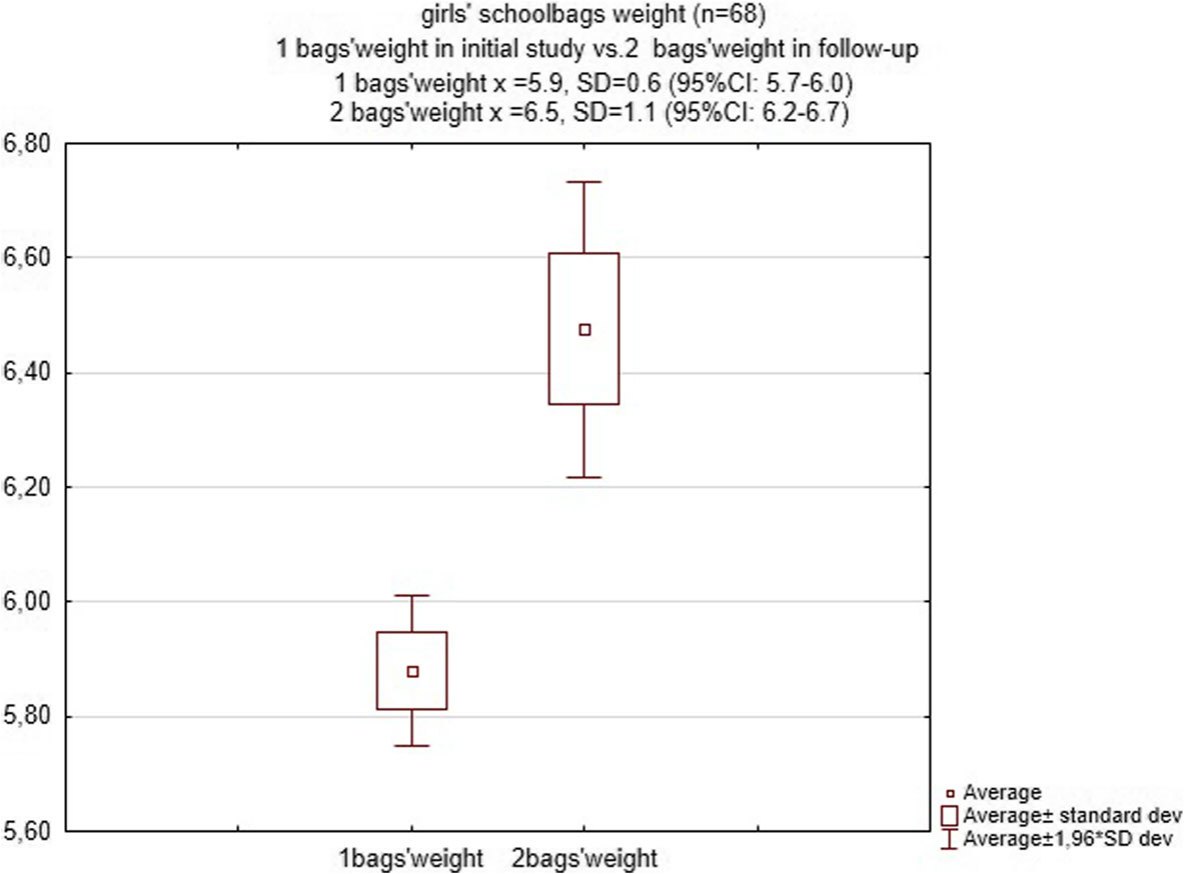
Average and standard deviation of the school bags’ weight in initial study and follow-up in girls group

**Fig. 4:**
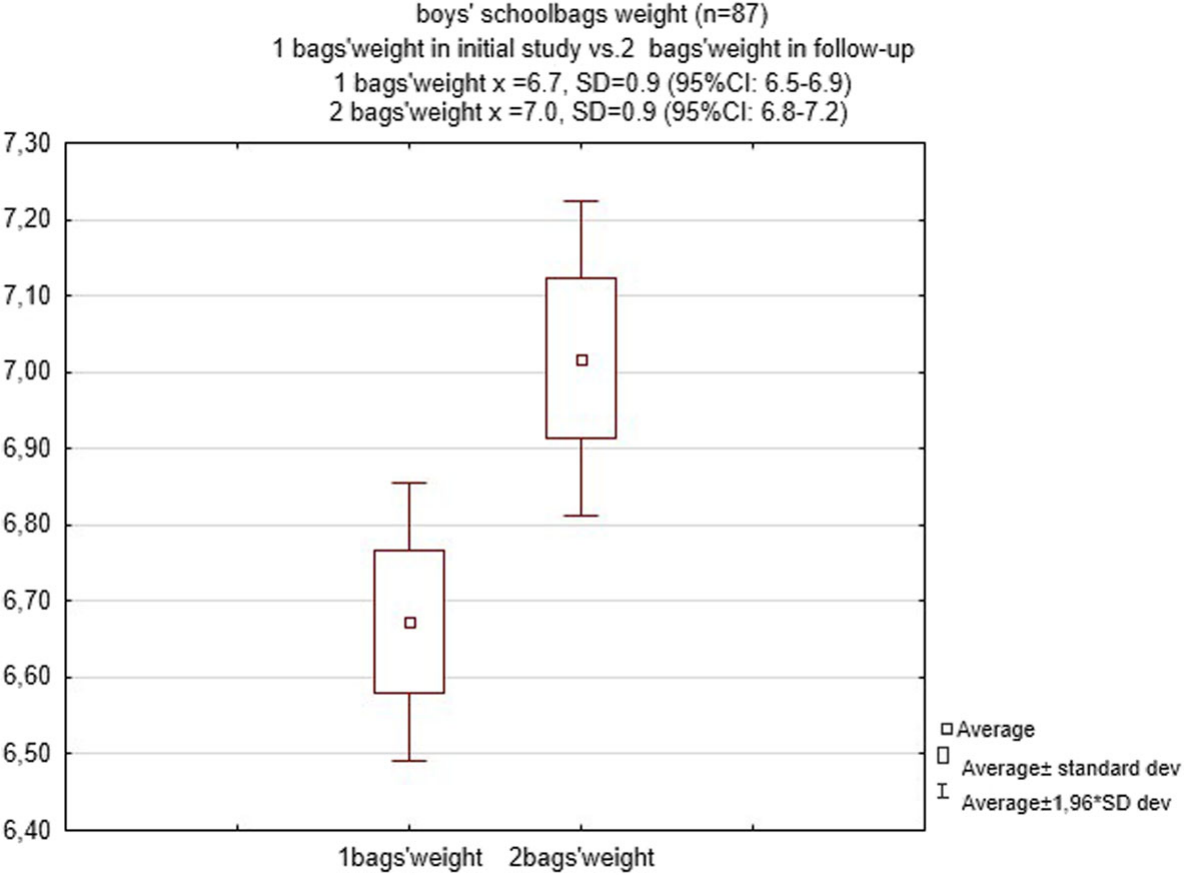
Average and standard deviation of the school bags’ weight in initial study and follow-up in boys group

The weight of a school bag in the second examination ranged between 4.7 and 9.8 kg (mean = 6.8 ± 1.05) and a statistically significant increase was observed in comparison to school bag weight in the initial examination (z = 5.6; *p* < 0.00001). There was an average increase of 1.3 ± 0.8 kg among 55.9% of girls and a decrease among only 17.9% of girls. Among boys, 45.97% revealed an average school bag weight increase of 1.1 ± 0.5 kg (Figs. 3 and 4).

The difference in school bag weight changed during the year and depended on age; among girls, there was a direct relationship between these factors (R Spearman =0.3, *p* < 0.007), and among boys, there was not an inverse relationship between these factors (*R* = −0.12, *p* > 0.24). As expected, school bag weight was not determined by the difference in heights (*p* > 0.22) or weights (*p* > 0.78) of the pupils.

Among both examined groups, the recommended school bag weight was still exceeded by 95.5% on average at the second examination. The difference between these groups was statistically significant (z = 5.1, *p* < 0.00001).

#### School bags use

More than 90% of the examined pupils improperly put on and took off their school bags. In general, 75% of school bags of girls and 69% of school bags of boys were adjusted to the proper dimensions (length – width) and were of good quality. Only five girls (7.3%) and 11 boys (12.6%) carried profiled backpacks instead of school bags – they were pupils in third grade.

The pupils have been walking to school in time from 3 to 30 min (mean = 10.1 ± 5.5). There are no differences between girls and boys (*p* > 0.68).

#### Straps of school bags

The school bag straps were too short for 15.5% of pupils and too long for 26.4%. Unfortunately, 79.3% of the pupils (83.8% of girls and 75.9% of boys) had the two straps adjusted to different lengths. Although this association was not statistically significant (*p* > 0.22), the asymmetry ranged between 1 and 9 cm among boys (mean = 3.1 ± 1.6) and between 1 and 9 cm among girls (mean = 3.3 ± 1.8 cm). The differences of straps length depended on age (*R* = 0.17, *p* < 0.032) but not on BMI (*R* = −0.15, *p* > 0.07). Differences in school bag straps lengths depended on pupil’s grade (Spearman R = 0.2, *p* < 0.04).

Asymmetry of school bag straps ranged from 2.5 to 9 cm in as many as 50.3% (girls: 55.9%; boys: 45.98%) of subjects (x = 4.2 ± 1.4 cm). Straps were significantly longer an average of 2.2 cm in second examination than in initial examination (t = 6.5, *p* < 0.0000001), although 40.6% of pupils still wore their straps at improper lengths (too long for 49.7% and too short for 34.0%). Strap lengths were shortened in the final examination among 37.7% of girls and 10.3% of boys, and strap lengths were lengthened among 34% and 71.3%, respectively. In the case of straps which were already too long, 27.9% of girls and up to 37.9% of boys lengthened them further (Table 4). In 22 of children (16.2% of girls and 13.8% boys) braces which have been balanced in the initial test became asymmetrical in the final study.

**Table 4:** Percentage values of the straps of school bags in stabilization, shortening and elongation according to sex

Subgroup sex	Shortening			Stabilization			Elongation		
	Shortness	Appropriate	Lengthy	Shortness	Appropriate	Lengthy	Shortness	Appropriate	Lengthy
	N (%)	N (%)	N (%)	N (%)	N (%)	N (%)	N (%)	N (%)	N (%)
F - 68	12 (17.6%)	15 (22.1%)	1(1.5%)	2 (2.9%)	11 (16.2%)	-	6 (8.8%)	14 (20.6%)	8 (11.8%)
M - 87	-	9 (10.3%)	-	4 (4.6%)	10 (11.5%)	3 (3.4%)	-	31 (35.6%)	30 (34. %)

Stabilization - no significant changes in the straps of school bags in the final examination - relative to a preliminary examination

Shortening - significant shortening changes in the straps of school bags in the final examination - relative to a preliminary examination (defined as: shortness, appropriate, lengthy)

Elongation - significant elongation changes in the straps of school bags in the final examination - relative to a preliminary examination (defined as: shortness, appropriate, lengthy)

*Abbreviations* **:** *F* female, *M* male

### Posturometric parameters

Relative to the initial examination, all groups in the second examination (final) revealed changes in posturometric parameters. Statistically significant differences were observed between examinations among girls and boys (Table 2). These result excluded torso rotation sum calculated by the Hump – Sum indicator, in which final and initial results were similar. However, rotations at particular levels were not comparable. Sex did not have any influence on the obtained results (*X* ^*2*^ = 10.6, *p* < 0.004, df = 2). Torso rotation increased and exceeded the recommendations in 35.29% of girls by an average of 2.7° ± 1.2° and in 60.9% of boys by an average of 22.3° ± 1.3°. Rotation values ranged between 4° and 6° in 51.7% of boys and 32.3% of girls; they were given information about posture abnormalities and they were told to repeat body posture examination within 6 months and referred to a physiotherapist. Rotation values for two girls and eight boys were above 7°; they were referred to an orthopedic specialist for further diagnosis. Kyphosis angle was greater than the Dobosiewicz norms for 48.5% of the girls and 36.8% of boys.

As children with growth spurts were excluded from the analysis, the results of the posturometric parameters did not depend on differences between two examinations (*p* > 0.08), with the exception of the shoulder blade positions, which were only significantly different between examinations among girls (*R* = 0.2, *p* < 0.007). Only 23.2% of children (27.9% girls and 19.5% boys) had postural muscles endurance of 30 s. It depended on age (*R* = − 0.32, *p* < 0.04) and on the weight of school bagsin girls (*R* = − 0.4, *p* < 0.03) and boys (*R* = −0.5, *p* <0.02). Postural muscles performance has not changed significantly over the annual observation (Table 2). Immutable values have been reported in 55.9% of girls and 50.6% of boys, while the deterioration has been observed respectively in 14.7% and 24.1%.

After 1 year, nearly 93.5% of pupils improperly put on and took off their school bags, often by performing a bending motion accompanied by rotation and rapid straightening of the lumbar spine.

### Factors associated with postural disorders

The age of boys correlated with the following posturometric parameters: rotation of the thoracic – lumbar spine (R Spearman =0.3, *p* < 0.004), projection of the plumb line from the gluteal cleft (*R* = 0.28, *p* < 0.0007) and shoulder blade position (R Spearman =0.2, *p* < 0.03). However, no similar relationships were observed among girls. The increase in straps length was significantly associated with decreased kyphosis angle (*R* = − 0.17, *p* < 0.02).

As the main inclusion criterion was proper body posture, it was not necessary to correlate school bag results with body posture results. These analyses were instead conducted after 1 year of observation. These results can be justified by biomechanical responses to moments acting on the center of gravity and force distribution.

Following factors have been taken into account in the analysis: differences in weight of a backpack, straps length, backpack orientation on the back of the children in both studies (differences between final test and initial test) and differences in measured parameters, taking into account normative values for each test. In the group of girls, backpack weight changes affect the vertical deviation and the main rotation components: thoracic spine and Hump factor. In boys, these changes correlated with rotation component in thoracic and thoracolumbar spine. Backpack straps asymmetry had an impact on changes in certain body posture parameters (depth of lumbar lordosis, rotation of the trunk in thoracic spine and Hump factor) only in the group of girls. Position of the backpack location on the back (too high, correct, too low) in girls, affected the depth of lordosis and kyphosis (Table 5). Girls had much more lightweight backpacks than boys from the same class, contrary to the straps asymmetry assessment according to which in the group of girls, straps were significantly longer than in boys (Table 3).

**Table 5:** Correlations between the changes in the weight of school bags, in straps length, positioning and changes in posturometric parameters in both groups after 1 year

Parameters	Sex	The differences of school bags between initial and follow-up of		
		Weight	Straps	Positioning on the back
Plumb – line anal cleft (cm)	*F*	0.22*	−0.006	−0.12
	*M*	0.19	0.14	0.08
Scapulae level (°)	*F*	−0.07	0.01	−0.08
	*M*	−0.06	−0.001	−0.0001
Kyphosis angle (°)	*F*	0.1	0.17	0.53*
	*M*	−0.05	0.13	−0.07
Lordosis angle (°)	*F*	0.1	0.23*	0.4*
	*M*	0.07	0.17	0.16
Angle of trunk rotation C_7_ – Th_1_ (°)	*F*	−0.12	0.08	0.01
	*M*	0.02	0.04	0.14
Angle of trunk rotation Th (°)	*F*	0.33*	−0.24*	−0.21
	*M*	0.23*	0.07	0.11
Angle of trunk rotation Th-L/L(°)	*F*	0.14	−0.01	−0.09
	*M*	0.24*	0.04	0.15
Sum of trunk rotation (HUMP SUM°)	*F*	0.23*	−0.34*	0.04
	*M*	0.11	−0.004	0.09
Mathiass test	*F*	−0.25*	−0.05	0.11
	*M*	−0.37*	0.01	−0.09

*Abbreviations* **:** *F* female, *M* male

*p < 0.05; *P* – value according to R Spearman

## Discussion

The purpose of the study was to evaluate body posture changes and describe their relationship with school bag characteristics in pupils aged 7–9 years. This study showed that children wear heavy school bags which mean weight in the initial study was 6.3 ± 0.8 (range between 4.7 and 9.0 kg). A tendency to carry slightly heavier school bags was noted in boys. The increase of torso rotation exceeding norms was observed in 35.2% of girls and in 60.9% of boys. The increase of kyphosis angle over norms indicated by Dobosiewicz was noted in 77.9% of girls and in 62.8% of boys.

Factors which could affect the variability of the results sought to be eliminated. The study included institutions (schools) which head classes with a fixed number of seven teaching hours every day throughout the school year. The division of subjects into particular days did not affect the weight of backpacks. In addition, very strict criteria for inclusion in the study allowed to eliminate factors which may affect the development of body posture. A shortcoming is the lack of reliable observation of children (ergonomic behaviors) on the way to school and back - therefore it was not a subject of the analysis.

Irrespective of the type of small deviations from the proper body posture, important negative changes were observed over the course of the year. Body posture is influenced by many endogenous and exogenous factors [23]. Because of the fact that endogenous factors were not always under our influence, exclusion and inclusion criteria were selected very precisely. One of those exclusion criteria was the evidence of a growth spurt [2, 3] because it is a period during which irregularities in body posture often appear [37, 38]. A growth spurt is caused by a rapid increase in bone length and is followed by certain limitations of muscular elasticity (especially of postural muscles) which enable this rapid growth [39]. The second important exclusion criterion was a long absence from physical education classes, most often because of allergies or frequent infections [40].

Reduced participation in physical activity [9, 12, 13], especially during this time of transition from a dynamic to a static lifestyle, may itself cause an imbalance in the tension of antagonist postural muscles and may lead to serious posture disorders with time [1]. Although there are some simple methods to address this problem [41, 42, 43], they require a great deal of adult control (parents, legal guardians or teachers) during early primary school [23]. In this study, such solutions and their influence on body posture and school bag weight have not been presented because results in this field greatly vary. Exogenous factors [44] may be strongly modified, e.g., by attaining a proper position during sitting, standing, learning or playing, by reducing school bag weight during early primary school, by adjusting school bags and by shortening straps which are too long.

The inversely proportional relationships of strap length to the depth of kyphosis and lumbar lordosis angle are understood from a biomechanical point of view, as they are the result of the distribution of moments and forces caused by the school bag load [45]. For instance, child attempting to balance a school bag placed too high on his/her back will lean forward and overload the spine [45, 46]. This adaptation leads to an imbalance of antagonist muscles, such that the torso muscle activity dominates as a functional effect [47]. When parents buy a school bag for their first grader, they consider its price, weight and the profile of the spine of the child and back of the school bag [23]. Previous work of the author confirmed that school bags were generally chosen appropriately but the pupils improperly adjusted their school bags or swapped school bags for an outdoor – style backpacks during third grade or higher.

School bag itself did not address all of the ergonomic aspects; school bags were most frequently placed too high, what forced children to lean forward and overload their spines [47, 48]. The overloading was then resolved by increased thoracic kyphosis and lumbar lordosis angles [19, 49, 50]. Another problem is that the maximum load approved for younger pupils is equal to 10–15% of their body mass [23, 50, 51]. This factor is not sufficient. Presented research showed that only 23% of examined children had a postural muscle endurance of 30 s. If the Matthias’s test position was held for less than 5 s, then the burden of the overloaded school bag would exacerbate this failure. This burden may lead to posture abnormalities. These results should prompt a broader discussion.

Due to the fact that these studies were observational and lasted a full school year, it was difficult to eliminate all of the confounding factors which may have a direct impact on changes in body posture. One of them is the growth spurt – but this factor has been completely eliminated. Probably, longer observation will allow to draw wide – ranging conclusions about the impact of the backpack (its weight, straps asymmetry, asymmetry straps, proper wearing and simple ergonomic principles) on the body posture especially in the higher grades. Evaluation of the same study group after cyclic training on ergonomics in the context of the author's program "my healthy spine” should be analyzed further.

The unsatisfying results of studies conducted over several years encouraged the author to introduce a prophylactic program “My healthy spine at school” in Poland [16, 23]. During practical classes, children learned about the structure and function of the spinal column, about proper and improper movement patterns when learning and playing, and about the causes of postural disorders and how they may affect their physical abilities as adults [16, 23]. Parents of the children were also instructed on how to create an ergonomic workplace for their children and how to constantly control an ideal body position. Detailed analysis of behavioral training aspects has been performed, and the weight of a school bag of the pupil was a significant factor to consider. The spine protection program is required on account of a sedentary lifestyle and the increasing rate of postural defects/deformations which occur during the development from youth to adolescence [16, 23]. Data from 6 years of practice have been collected for further research and analysis – maybe these data will provide an ideal strategy to diminish or even eliminate the effects of modifiable exogenous factors of body posture irregularities.

## Conclusions

School bags of the pupils were heavier than the recommendation in 79% of boys and in 64% of girls. Weight differences of the school bags after one school year have influenced changes in body posture abnormalities, especially in rotation parameters. Backpack straps asymmetry is noticeably stronger in the group of girls and the difference between braces may have an impact on posturometric parameters. Lack of proper backpack lifting skills tends to create programs and training systems in this regard.

### Implications for practice

Due to increasingly sedentary lifestyle and the increase in posture abnormalities, as well as spinal deformations during psychomotor development, there has been a greater emphasis on school bag weight. Parents and teachers must be engaged until an appropriate practice is formed, i.e. in order to always unpack unnecessary things and to pack only necessary objects for a particular day. Furthermore, there is a need to rethink postural positions and ergonomic behaviors with school bags carried every day.
